# Regulation and functional roles of rebound potentiation at cerebellar stellate cell—Purkinje cell synapses

**DOI:** 10.3389/fncel.2014.00042

**Published:** 2014-02-18

**Authors:** Tomoo Hirano, Shin-ya Kawaguchi

**Affiliations:** ^1^Department of Biophysics, Graduate School of Science, Kyoto University Kitashirakawa-Oiwake-choKyoto, Japan; ^2^Graduate School of Brain Science, Doshisha UniversityKyoto, Japan

**Keywords:** cerebellum, Purkinje cell, synaptic plasticity, rebound potentiation, long-term potentiation, motor learning, inhibitory synapse, GABA

## Abstract

Purkinje cells receive both excitatory and inhibitory synaptic inputs and send sole output from the cerebellar cortex. Long-term depression (LTD), a type of synaptic plasticity, at excitatory parallel fiber–Purkinje cell synapses has been studied extensively as a primary cellular mechanism of motor learning. On the other hand, at inhibitory synapses on a Purkinje cell, postsynaptic depolarization induces long-lasting potentiation of GABAergic synaptic transmission. This synaptic plasticity is called rebound potentiation (RP), and its molecular regulatory mechanisms have been studied. The increase in intracellular Ca^2+^ concentration caused by depolarization induces RP through enhancement of GABA_A_ receptor (GABA_A_R) responsiveness. RP induction depends on binding of GABA_A_R with GABA_A_R associated protein (GABARAP) which is regulated by Ca^2+^/calmodulin-dependent kinase II (CaMKII). Whether RP is induced or not is determined by the balance between phosphorylation and de-phosphorylation activities regulated by intracellular Ca^2+^ and by metabotropic GABA and glutamate receptors. Recent studies have revealed that the subunit composition of CaMKII has significant impact on RP induction. A Purkinje cell expresses both α- and β-CaMKII, and the latter has much higher affinity for Ca^2+^/calmodulin than the former. It was shown that when the relative amount of α- to β-CaMKII is large, RP induction is suppressed. The functional significance of RP has also been studied using transgenic mice in which a peptide inhibiting association of GABARAP and GABA_A_R is expressed selectively in Purkinje cells. The transgenic mice show abrogation of RP and subnormal adaptation of vestibulo-ocular reflex (VOR), a type of motor learning. Thus, RP is involved in a certain type of motor learning.

## Introduction

**Figure 1:**
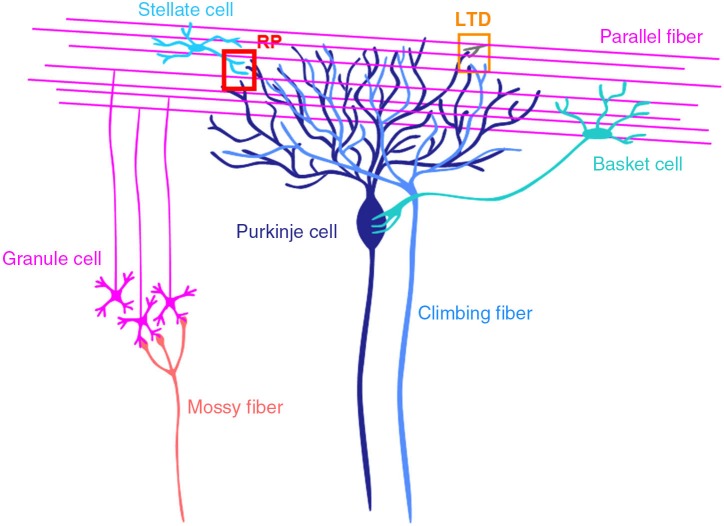
**Cerebellar cortical neuronal circuits.** Mossy fibers from pontine nuclei etc., send excitatory synaptic outputs to granule cells. A granule cell forms one or a few excitatory glutamatergic synapses on a Purkinje cell, where LTD occurs depending on the activity of the granule cell and a climbing fiber. Molecular layer interneurons (stellate and basket cells) receive excitatory synaptic inputs from granule cells and inhibit Purkinje cells. At inhibitory GABAergic synapses between a stellate cell and a Purkinje cell, rebound potentiation (RP) is induced by climbing fiber activity.

The cerebellum consists of cortex and nuclei, and is involved in motor control (Figure 1; Ito, 1984, 2011; Llinás et al., 2004). There are two major inputs to the cerebellum, mossy fibers and climbing fibers. Mossy fibers coming from pons, medulla oblongata etc., innervate neurons in cerebellar nuclei and granule cells in the granular layer of cortex. Granule cells extend axons to the molecular layer, where they bifurcate. The bifurcated granule cell axons are called parallel fibers, and form excitatory glutamatergic synapses on dendrites of Purkinje cells and inhibitory GABAergic interneurons in the molecular layer, stellate and basket cells. Climbing fibers coming from inferior olivary nuclei innervate neurons in cerebellar nuclei and Purkinje cells. A single climbing fiber forms hundreds synapses on a Purkinje cell, and thus sends a powerful excitatory drive. Purkinje cells are GABAergic neuron, and send sole output from the cortex to nuclear neurons.

Climbing fibers are thought to code error signals (Maekawa and Simpson, 1973), and regulate activities of Purkinje cells. Activation of parallel fibers followed by activation of a climbing fiber depresses the efficacy of synaptic transmission between the activated parallel fibers and a Purkinje cell long-term. This synaptic plasticity is called long-term depression (LTD), and has been considered to be a cellular basis of motor learning such as adaptation of reflex eye movements and classical conditioning of eye blink response (Ito, 1982, 2011; du Lac et al., 1995; Thompson, 2005; Hirano, 2013a). However, mice defective in LTD were shown to display normal motor learning (Welsh et al., 2005; Schonewille et al., 2011), and the involvement of other plasticity mechanisms in motor learning has been suggested (Hansel et al., 2001; Jörntell and Hansel, 2006; Dean et al., 2010; Jörntell et al., 2010; Gao et al., 2012; Hirano, 2013a).

**Figure 2:**
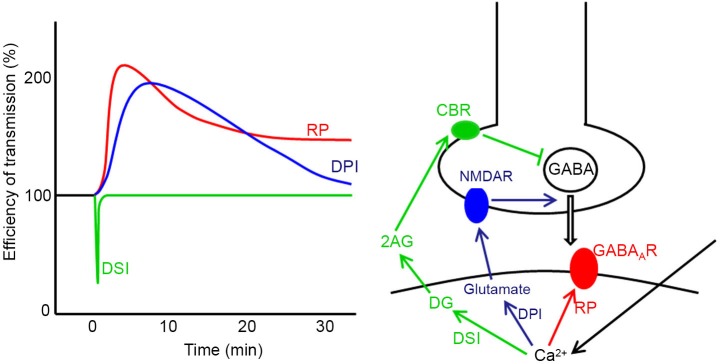
**Three forms of synaptic plasticity at stellate cell—Purkinje cell synapses.** Time courses (left) and induction mechanisms (right) of DSI, DPI and RP are presented. In DSI the Ca^2+^ increase caused by postsynaptic depolarization produces diacylglycerol (DG), which is broken down to 2-arachidonylglycerol (2AG). 2AG reaches the presynaptic terminal and activates cannabinoid receptor 1 (CBR) on the cell membrane, which suppresses presynaptic vesicular release of GABA. In DPI, the intracellular Ca^2+^ increase causes postsynaptic release of glutamate, which activates presynaptic NMDA receptor (NMDAR) potentiating presynaptic GABA release. In RP, the postsynaptic Ca^2+^ increase potentiates postsynaptic GABA_A_R responsiveness.

Plasticity also takes place at synapses other than parallel fiber-Purkinje cell synapses in the cerebellum such as excitatory synapses on granule cells, those between parallel fibers and inhibitory interneuron and those in the nuclei (Jörntell and Ekerot, 2002, 2003; D’Angelo et al., 2005; Pugh and Raman, 2006). At GABAergic synapses formed by stellate cells on Purkinje cells, three types of plasticity induced by postsynaptic depolarization have been reported (Figure 2), namely, depolarization-induced suppression of inhibition (DSI), depolarization-induced potentiation of inhibition (DPI) and rebound potentiation (RP) (Hirano, 2013b). DSI is short-lasting suppression of presynaptic GABA release mediated by endocannabinoid, which is released from a Purkinje cell and binds to presynaptic cannabinoid receptor (Llano et al., 1991; Yoshida et al., 2002). DPI is longer-lasting potentiation of presynaptic GABA release mediated by glutamate, which is released from a postsynaptic Purkinje cell and binds to presynaptic NMDA receptors (Duguid and Smart, 2004). RP occurs postsynaptically and lasts longer (Kano et al., 1992; Kawaguchi and Hirano, 2000; Tanaka et al., 2013). In RP, postsynaptic responsiveness to GABA is enhanced. These plasticity mechanisms are triggered by the postsynaptic Purkinje cell depolarization and subsequent intracellular Ca^2+^ increase (Figure 2). Thus, they are hetero-synaptic plasticity induced by excitatory inputs. In this article, molecular regulatory mechanisms of RP induction and functional roles of RP are reviewed.

## Mechanism of rebound potentiation (RP) induction

RP is induced by activation of a climbing fiber or direct depolarization of a postsynaptic Purkinje cell that causes large increase in the intracellular Ca^2+^ concentration [Ca^2+^]_*i*_ (Kano et al., 1992; Miyakawa et al., 1992). Stimulation of a climbing fiber five times at 0.5 Hz induces RP in juvenile cerebellar slice preparations (Kano et al., 1992). However, in that study an intracellular solution containing high concentration of Cs^+^ was used, and subsequent studies used direct depolarization of a Purkinje cell (Kano et al., 1992; Kawaguchi and Hirano, 2000, 2002, 2007; Kitagawa et al., 2009; Tanaka et al., 2013). Thus, patterns of climbing fiber activity sufficient to induce RP *in vivo* remain unclear. The time integral of [Ca^2+^]_*i*_ is correlated with the induction of RP, and RP is induced in an all-or-none fashion with a certain threshold (Kitagawa et al., 2009; Kawaguchi et al., 2011). RP has been monitored with the amplitude of inhibitory postsynaptic current or that of Cl^−^ current induced by GABA applied to dendrites, and it has been shown that RP is expressed as enhanced postsynaptic responsiveness to GABA (Kano et al., 1992; Kawaguchi and Hirano, 2000, 2002, 2007). Stellate cells form inhibitory synapses on dendrites, whereas basket cells form them on the soma of a Purkinje cell. RP has been studied primarily at stellate cell—Purkinje cell synapses in dendrites. Whether RP occurs similarly at basket cell—Purkinje cell synapses is unclear. It was difficult to record RP when GABA was applied to a soma (our unpublished observation). However, this difficulty might have been ascribed to washout of intracellular molecules necessary for RP induction caused inadvertently by the whole-cell recording conditions.

Increased intracellular Ca^2+^ binds to calmodulin, which in turn binds to Ca^2+^/calmodulin-dependent kinase II (CaMKII). CaMKII activity is necessary for RP induction (Kano et al., 1996; Kitagawa et al., 2009). CaMKII is known to phosphorylate many proteins including GABA_A_R β and γ2 subunits (Moss and Smart, 1996; Brandon et al., 2002; Houston et al., 2009). Purkinje cells express α1, β2, β3 and γ2 subunits which form a heteropentameric GABA_A_R, and β2 is more abundant than β3 (Laurie et al., 1992; Wisden et al., 1996; Pirker et al., 2000; Hirano, 2013b). Houston et al. (2008) reported the CaMKII mediated increase in IPSC amplitudes in cerebellar granule cells expressing GABA_A_R containing β2 subunit. Thus, direct phosphorylation of β2 subunit of GABA_A_R by CaMKII could be involved in RP. However, it was also reported that CaMKII potentiates α1β3γ2 GABA_A_R but not α1β2γ2 receptor in undifferentiated NG108-15 neuroblastoma cells (Houston and Smart, 2006), suggesting that the potentiation of β2 subunit-containing GABA_A_R by CaMKII may not work in some conditions or in certain cells (Houston et al., 2009). Thus, roles of direct phosphorylation of GABA_A_R by CaMKII in RP remain enigmatic.

Another target molecule of CaMKII in RP induction is GABA_A_R associated protein (GABARAP). GABARAP has a binding site for GABA_A_R γ2 subunit (Wang et al., 1999). RP induction is impaired by competitive inhibition of association between GABARAP and GABA_A_R γ2 subunit with a peptide (γ2 peptide) corresponding to the intracellular region of γ2 subunit that mediates the binding to GABARAP (Kawaguchi and Hirano, 2007). Application of this peptide after establishment of RP also attenuates once-established RP, suggesting that the interaction of GABARAP and γ2 subunit is required not only for induction of RP but also for its maintenance. Fluorescence resonance energy transfer (FRET) imaging experiments showed that GABARAP undergoes a sustained structural change in response to depolarization of a Purkinje cell (Kawaguchi and Hirano, 2007). This conformational change of GABARAP depends on activity of CaMKII. Further, single amino acid replacement of GABARAP V33E blocks structural change of GABARAP and suppresses RP induction. Thus, CaMKII-mediated conformational change of GABARAP seems to be essential for RP. GABARAP is involved in intracellular trafficking and targeting of GABA_A_R to the cell membrane (Kneussel et al., 2000; Kittler et al., 2001; Moss and Smart, 2001; Kneussel, 2002; Nymann-Andersen et al., 2002; Leil et al., 2004; Lüscher and Keller, 2004; Chen and Olsen, 2007; Kanematsu et al., 2007). Thus, GABARAP might induce RP through facilitating GABA_A_R transport to the cell membrane. In hippocampal neurons, inhibitory synaptic potentiation is induced by activation of NMDA-type glutamate receptors through GABARAP-dependent exocytosis of GABA_A_R (Marsden et al., 2007). Another possible role of GABARAP in RP is to enhance the function of individual GABA_A_R by increasing the single channel conductance or the open time (Everitt et al., 2004; Luu et al., 2006). GABARAP is also known to bind to tubulin, and it has been suggested that association of GABARAP with tubulin is required for RP induction (Kawaguchi and Hirano, 2007).

## Mechanism of rebound potentiation (RP) suppression

RP is induced by cell-wide depolarization of a Purkinje cell caused by hetero-synaptic excitatory climbing fiber inputs (Kano et al., 1992). Thus, RP should occur at many inhibitory synapses on a Purkinje cell simultaneously, and should not be synapse-specific. However, there is a synapse-specific regulatory mechanism for RP induction. GABAergic synaptic transmission or GABA_B_ receptor activation during the postsynaptic depolarization suppresses RP (Kawaguchi and Hirano, 2000). This regulation is unique in that homo-synaptic activity suppresses induction of synaptic plasticity. Usually, homo-synaptic activity triggers the plasticity of transmission.

**Figure 3:**
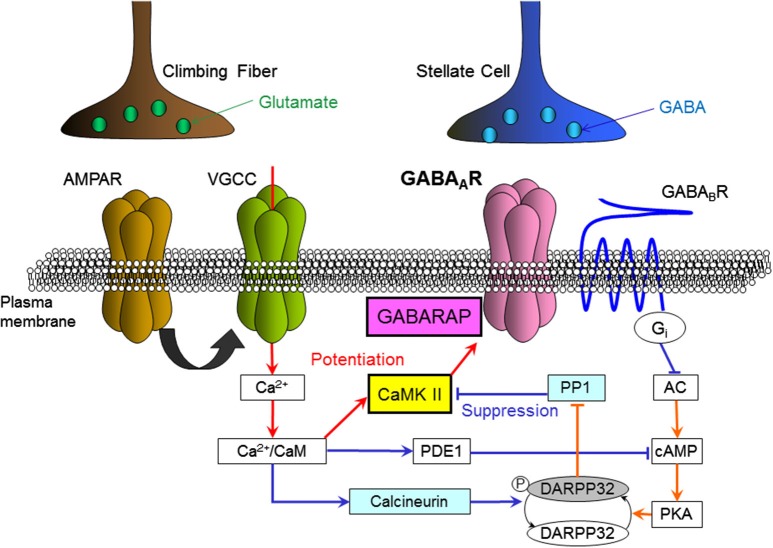
**Intracellular molecular signaling cascades regulating RP.** Arrows indicate an increase, activation or enhancement, and T-bars indicate a decrease or suppression. Red lines indicate signal transmissions which work to induce RP, and blue lines indicate those work to suppress RP. AMPAR, AMPA-type glutamate receptor; VGCC, voltage-gated Ca^2+^ channel; GABA_A_R, GABA_A_ receptor; GABA_B_R, GABA_B_ receptor; CaM, calmodulin; CaMKII, Ca^2+^/calmodulin-dependent kinase II; GABARAP, GABA_A_R associated protein; PDE1, phosphodiesterase 1, PP1, protein phosphatase 1; DARPP32, dopamine and cAMP-regulated phospho-protein 32 kDa; PKA, protein kinase A; cAMP, cyclic-adenosine-monophosphate; AC, adenylyl cyclase; Gi, Gi protein.

This GABA_B_ receptor-dependent suppression of synaptic plasticity is mediated by down-regulation of the activity of protein kinase A (PKA). It was revealed that down-regulation of PKA activity decreases the amount of phosphorylated dopamine- and cyclic adenosine monophosphate (cAMP)-regulated phospho-protein 32 kDa (DARPP-32; Kawaguchi and Hirano, 2002; Figure 3). Phosphorylated DARPP-32 is known to inhibit protein phosphatase 1 (PP1), which de-phosphorylates CaMKII and other phosphorylated proteins (Greengard et al., 1999). Thus, GABA_B_ receptor activation works to enhance PP1 activity counteracting CaMKII. It was also shown that a Ca^2+^-dependent phosphatase calcineurin de-phosphorylates DARPP-32 upon a [Ca^2+^]_*i*_ increase and supports suppression of RP (Kawaguchi and Hirano, 2002). A later study showed that the basal PKA activity in a Purkinje cell is partly supported by the activity of metabotropic glutamate receptor mGluR1 (Sugiyama et al., 2008).

## Signaling cascade regulating rebound potentiation (RP)

The preceding sections have introduced molecules involved in regulation of RP. Among them CaMKII is a key molecule for RP induction. There are two subtypes of CaMKII, α and β, and the relative expression level of β-CaMKII to α-CaMKII is higher in the cerebellum than in the forebrain (McGuinness et al., 1985; Walaas et al., 1988). In the cerebellar cortex β-CaMKII is expressed in several types of cells including Purkinje cells, whereas α-CaMKII is expressed only in Purkinje cells. It has been reported that the relative amounts of α- and β-CaMKII change depending on the neuronal activity and developmental stage in the mammalian central nervous system (Bayer et al., 1999; Thiagarajan et al., 2002). β-CaMKII has much higher affinity to Ca^2+^/calmodulin than α-CaMKII (Brocke et al., 1999). In addition β-CaMKII binds to actin but α-CaMKII does not (Okamoto et al., 2009). Thus, subtypes of CaMKII may have different roles in a Purkinje cell. Recently, we addressed this point by overexpressing or knocking-down each type of CaMKII, and found that the subunit composition of CaMKII has a significant impact on RP induction (Nagasaki et al., 2012). Suppression of the expression of β-CaMKII but not that of α-CaMKII inhibits RP induction, whereas overexpression of α-CaMKII but not that of β-CaMKII inhibits the induction. Thus, the relative amount of β- to α-CaMKII seems to be critical for RP induction.

Interactions among molecules regulating RP including CaMKII are complex, as there are multiple branchings and feedback loops in the signaling cascades (Figure 3). Thus, it is difficult to intuitively predict how they behave quantitatively. To address this question, a theoretical model of molecular signaling networks for RP regulation has been built and computational simulation has been performed (Kitagawa et al., 2009; Kawaguchi et al., 2011). During this process phosphodiesterase 1 (PDE1), a Ca^2+^/calmodulin-dependent enzyme that breaks down cAMP, was added as a critical element. The simulation reproduced essential features of induction and suppression of RP, and suggested that PDE1 plays a predominant role in determination of the Ca^2+^ threshold for RP induction (Kitagawa et al., 2009). Regulation of RP induction by a cell adhesion molecule integrin was also reported (Kawaguchi and Hirano, 2006).

## Ca^2+^ context regulates rebound potentiation (RP)

RP induction depends on leaky integration of the intracellular Ca^2+^ concentration (Kawaguchi et al., 2011) as induction of LTD at glutamatergic parallel fiber—Purkinje cell synapses does (Tanaka et al., 2007). However, it is not just integration of the Ca^2+^ signal that is critical for RP induction. We found that the context or order of the Ca^2+^ signal affects RP induction (Kawaguchi et al., 2011). Either a large and short increase in the intracellular Ca^2+^ concentration, or a small and long one can induce RP by itself. However, when a large and short increase is followed by a small and long increase, RP is not induced. In contrast, when the order is reversed, RP is induced. Thus, RP induction depends on the context or the time course of intracellular Ca^2+^ change. It was suggested that this interesting context-dependence of RP induction on the intracellular Ca^2+^ concentration is brought about by context-dependent autophosphorylation at Thr305/306 of CaMKII, which negatively regulates the subsequent Ca^2+^/calmodulin-dependent activation of CaMKII (Figure 3).

## Involvement of rebound potentiation (RP) in motor learning

Until recently there was no experimental evidence about roles of RP in cerebellar functions. We thought that RP might work together with LTD for establishment of motor learning, because activation of an inferior olivary neuron contributes to induction of both LTD and RP (Kano et al., 1992; Ito, 2011; Hirano, 2013a), and also because both down-regulation of excitatory synaptic inputs by LTD and up-regulation of inhibitory synaptic inputs by RP should work to suppress activity of a Purkinje cell. To test this idea, we generated transgenic mice defective in RP (Tanaka et al., 2013). As explained above, binding of GABA_A_R and an intracellular protein GABARAP is necessary for RP induction, and γ2 peptide which blocks this binding suppresses the induction. Transgenic mice which express γ2 peptide fused to a fluorescent protein only in Purkinje cells were generated. The transgenic mice do not show RP as we expected, and other physiological and morphological properties of the cerebellum including LTD induction appear normal.

**Figure 4:**
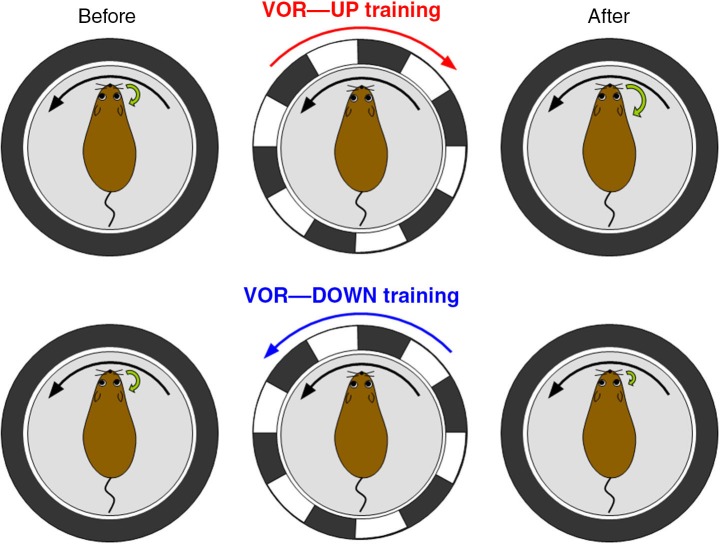
**Vestibulo-ocular reflex (VOR) and its adaptation.** VOR is induced by rotating a turntable on which a mouse is fixed in the dark. In VOR eyeballs turn in the opposite direction of head turn. VOR undergoes adaptive modifications. When a wild-type mouse and a surrounding screen with vertical black and white stripes are rotated in opposite directions in the light (VOR-up training), the gain of VOR increases gradually. In contrast, when the rotations are in the same direction (VOR-down training), the gain decreases.

Then, we evaluated the motor control and learning ability of the transgenic mice by examining reflex eye movement, vestibulo-ocular reflex (VOR; Figure 4). VOR is a reflex to turn an eyeball in the opposite direction of head turn, and works to stabilize visual image during head motion (Robinson, 1981). VOR undergoes adaptive modification in the direction to reduce image slip on a retina, which has been regarded as a model paradigm of cerebellum-dependent motor learning (Ito, 1982, 2011; Nagao, 1989; Lisberger et al., 1994; du Lac et al., 1995; Hirata and Highstein, 2001; Katoh et al., 2005; Hirano, 2013a). In experiments, a mouse is rotated sinusoidally on a rotating table, and a surrounding external screen with vertical black and white stripes is also rotated simultaneously (Tanaka et al., 2013). When the screen rotation is in the opposite direction to mouse rotation, the gain of VOR increases gradually in a wild-type mouse, and when the rotation is in the same direction, the gain decreases. These changes of VOR in a wild-type mouse are in the direction to reduce image motion on a retina and adaptive (Figure 4). These adaptive modifications of VOR amplitudes are suppressed in the transgenic mice defective in RP. Thus, transgenic mice defective in RP show defects in a type of motor learning, indicating that RP contributes to motor learning. However, it should be noted that these results do not rule out a possible contribution of LTD or other plasticity to motor learning. Indeed, adaptation of optokinetic response, another type of reflex eye movement, and reduced VOR adaptation occur in the RP-deficient mice (Tanaka et al., 2013). Considering similarities in induction conditions (Kawaguchi and Hirano, 2013) and suppressive effects on Purkinje cell activity between RP and LTD, they might synergistically support motor learning.

## Conclusion

Postsynaptic depolarization of a cerebellar Purkinje cell induces long-term potentiation (LTP) of GABAergic inhibitory synaptic transmission which is called RP. Induction of RP depends on Ca^2+^, CaMKII, GABARAP etc., and intricate regulatory mechanisms have been delineated. Transgenic mice defective in RP show defects in adaptation of VOR, indicating involvement of RP in motor learning.

## Conflict of interest statement

The authors declare that the research was conducted in the absence of any commercial or financial relationships that could be construed as a potential conflict of interest.
